# Bible and Business

**Published:** 1878-09

**Authors:** 


### BIBLE AND BUSINESS.

A merchant from New England came into our office yesterday, so feeble and thin that he almost staggered as he took his weary steps. He was fifty-two years old, laboring under both bodily and mental suffering quite sufficient to crush any man of ordinary spirit. By long years of close attention to business from the early morning until long hours after nightfall and by the steady practice of rigid economies and self-denials he had arrived at that point where he almost felt himself able to say, "Now I will give up the push of business and live upon the interest of my accumulations;" he thought his work was done and that now he would take his rest, and to that end had sold out his business at a handsome profit, when one bright morning of the beautiful May, an old citizen whom he had known as a man of position and wealth for thirty years called upon him and requested his name on a bond; in the confidence of citizenship and a neighborly acquaintance of more than thirty years the name was given. In thirty days the man failed and could not pay two cents on the dollar; to pay this bond swept away the entire fortune of our patient, as clear as the palm of the hand, and now old and poor and without a home, he was on his way to the recesses of the far West to seek a temporary shelter in the house of a distant relative, until he could compose his mind sufficiently to bring it to a contemplation of what was to be done for the future.

"I don't mind it so much for myself," said he, in a faltering accent, "but when I think of the long years of economy and self-denial and patient labor of my wife, it almost kills me; and then this horrid disease is so crushing out my life that I have not the energy to begin anew."

Now to see a grown man on the whimper line is a very considerable trial of our patience and yet we so deeply sympathyzed with him that before we knew it we had bounded up on our feet and was standing square in his front looking him straight in the eye making the following deliverence: "My good fellow, never say 'die,' because you are not going to do it for a 'considerable spell,' as you Yankees say; and giving up never did any good. Your lungs are as sound as a silver dollar, and with such a foundation to build upon, and such ailments as you have you are not likely to die these twenty years; and that is time enough for any man of moderate gumption to make two or three fortunes in these days. Have you never read in my JOURNAL about the little boy, not ten years old, slender, ragged, without a hat or shoe, trudging homeward by the Washington Monument in Union Square just at dusk of a cold snowy sloppy day in December, with a stick of wood as large as he could stagger under, on his shoulder, which he was carrying home to his widowed mother; it was very slippery, at last he fell sprawling in the slosh and a squall and a cry were naturally expected, but he jumped up in a moment, eyed the huge stick an instant and then exclaimed, 'Ding it, I'll try again,' and without more ado, he bowed his shoulder to it, and the next moment was trudging onward with more determination and a steadier step than before he fell." In an instant the poor man broke out into a deep hearty laugh, and his eyes so sparkled and twinkled that he seemed to grow twenty years younger, in a moment of time, as if he saw for himself a new success in the future. One of his friends had said to him, "How came you into such a box as this, a man known to be so prudent, so thrifty, so industrious and so attentive to business. I would have thought you would refuse to endorse for Wm. B. Astor." He could not answer the question. But many a man can remember in his own experience, that in some unaccountable way he has been surprised into a deed which was contrary to the principles and practices of a life time — surprised as it were, into some great fault, a most striking exemplification of the Scripture proverb, "He that trusteth in his own heart, is a fool," He that hateth suretyship is sure," (Prov. 11. 15) and that is the Bible method of doing a 'safe' business, and that it might not be forgotten, the wise man writeth a little futher on, 22. 26 — 'Be not thou one of them — that are sureties for debts.'

"Wonder if the fifteenth verse of the seventh chapter of Proverbs is not descriptive of the result of 'going security;' 'Therefore shall his calamity come suddenly, suddenly shall he be broken without remedy.' 'My son,' saith the first verse of these fifteen, 'if thou be surety for thy friend—thou art snared with the words of thy mouth.' Now what are the words of a man's mouth when he goes security for another? why there is not one in a thousand whose thoughts do not run in this wise, 'O, he's safe; there is no danger of my ever having to pay that debt.' How wise then is the Scripture saying of a suretyship, 'Thou art snared with the words of thy mouth.'"

Be assured, reader, that the Bible principles of doing business are best; and we will just step a mite out of our way to say a word for those whom we hire to work for us, what a saving of trouble and disappointment — heart-breaking disappointment it would be sometimes, many times, millions of times every day — if the Scripture injunctions were adhered to, the general idea being, that the poor should be paid for their personal service at the close of the day or at the time the work is completed or delivered. It was stated in the public papers that one of the most beautiful articles of apparel worn by the Queen of England at her coronation was made by a young woman, and that in walking a long distance, not for the first time either, on an inclement day, she died of a cold and the combined effects of bodily exhaustion and disappointment at not getting her pay, from the agents of the Sovereign; she of course could know nothing of such minor details. But how often tailors and poor seamstresses pine in actual want for work done for those who are revelling in the dance or the banquet-hall, it would be difficult to figure out. It often happens to a physician that he shall have a singular case, the first in a dozen years, and another, and even a third, within a day or two. It was not two weeks ago when a gentleman came into our office who had occupied a government position abroad for a number of years. We had known him here a score of years before in New York, as many others knew, to be one of our most active, enterprising and substantial business men. He was at the head of a house, Bank director, and a leading man in the charities of the times. He was the model of a generous-hearted, high-minded man, the soul of integrity and personal honor. In the crises of 1857 he failed, utterly; gave up every dollar, and with clean hands and an empty pocket struck out anew for business success; while doing so the wife of his youth died; his manly son perished in the ranks of the soldiers of the War; still he accumulated something handsome, and bereaved and old he came to New York to pass the short remnant of his days among the friends and scenes of his childhood. He was invited to make his home at the elegant mansion of a near relative, doing business in Wall Street, and of good repute. My friend said to him one day, "Here, I have some money; if in the prosecution of your business you find an opportunity of doing something to our mutual advantage, do so and divide the profits between us." In a short time his relative reported a good opportunity in a regular business transaction; said he, "A business house, Messrs. B., whom you know, offer to take from me every day so many thousand of a manufacture, at such a rate, to be paid for on delivery. I can get them made for such an amount; you see it is a necessary article of commerce; you know the parties who make and take; you see yourself the liberal margin between cost and price, and that there can be no mistake and no risk." This seemed fair, plain, honorable, without any possible risk. He was instructed to "go ahead." In less than a week he failed, utterly; there was not a dollar for any creditor. My friend made an investigation; he called on both manufacturer and purchaser; they were all right, but they had never seen his relative and had made no such engagements with him or any body else. The whole story was a fabrication from beginning to end, of an unprincipled man on the verge of bankruptcy.

Does not the reader's blood boil with rage at such conduct? A man inviting his relative to his own table and in all the confidence of guest and host and relationship, deliberately, day after day, carrying out purposes of deception and fraud, which it would seem no man in christendom could possibly have the effrontery to fabricate, and yet it was done; the whole thing is true to the last syllable.

The aggrieved man could not possibly have read his Bible to purpose, else he would have reasoned thus: "If a man who trusts himself is a fool, he who trusts another is a fooler."

The inspired page said of the Savior that he "did not commit himself unto them; for he knew what was in man." And yet the keenest, shrewdest men in business life are constantly found "napping."

A friend of ours made a purchase of some kegs of merchandise of one whom he met on "change" every day, and had done so for years. The key of the store-house was given him to examine them for himself. He sent one of his men to see if all was right, who returned and reported "all right," but not satisfied he sent his confidential clerk, who reported that he had examined a number of the kegs — that every one was of a superior brand — that the assortment was well packed to the very door — that he examined every one within his reach, and that all were alike — were A, number 1, and no mistake. The next day the seller was not on 'Change, nor the next, but it was of no consequence; my friend had paid fifty thousand dollars — was proud of the purchase — had the key in his pocket and he sent for his property; there was the apartment all locked, all safe; there were the identical kegs which had been examined, and the apartment was as full as ever; but there was one little thing in the way; all the kegs in the rear of the tier which had been examined had nothing in them but the bung-hole; so the fifty thousand dollars were gone at a clip and so was the seller, who, it was afterwards learned, was carried by remorse into an insane Asylum, in Switzerland.

Was not good old Jeremiah right when he said (17 v.) "Curseth be the man that trusteth in man." Within a month a good old elder in the church, whom we had known to be rich from our youth, came into our office and abruptly said, "Doctor, if I can't get help I and my family will be turned into the street next week, for I am a beggar." A short story was that, and a terrible. A man of sixty years of age with a wife and a large family of children from three to thirty, without a shelter and without a dollar. The character which his pastor gave of him was beautiful; it was worthy of being proud of, and to be hung on the wall framed in silver; and more, we know it to be true. But how did this sudden calamity come about? He was getting old, had prospered in business and had sold out to his younger partner, received all his money, deposited with his banker, whom he had known for years and years, to remain with him until he should determine its disposition. Within fifteen days that banker failed, paying fifteen cents to priviledged creditors. The Elder, full of confidence in his integrity and business capacity, with the promptness and selfreliance of a true business man, determined at once to obtain the situation of a salesman or clerk, which would keep the wolf from the door, not doubting that the wholesale houses with which he had had large and honorable dealings for many years would be glad to avail themselves of his abilities. Up and down Broadway and Maiden Lane and Wall Street he traveled day after day making a frank full statememt; not a man would employ hm; he had sympathy, codolence and courtesy, but no engagements. He next went to the friends of his youth, to advance a small sum each, which combined would start him in business, but not a dollar could he raise; "they all with one consent began to make excuse," and at the end of all he came to us, as above stated.

He will be rich again in money, as well as in christian character and business ability and integrity.

Who shall not say in the light of these suggestive narrations, coming under our own personal knowledge, that the Bible is a good business guide

"Go no man's security." "Trust no man." "Never give up — it don't do any good."

It is bad enough to be poor — it is worse to be in bad health, but to be old and poor and sickly, is terrible. Hard enough it is, for the great multitudes to get along in the world even when in the full enjoyment of bodily vigor, but to enter on the strife for bread under the crushing influence of poverty and disease is terrible to think of; and to avoid calamities so great, let every one read the Bible with greater care and practice more assiduously its lessons of wisdom and truth, for in this practice there is length of days and honor and peace in the life that now is, and in the world to come, life everlasting. The summing up of the whole article is founded, too, on Bible authority as the surest wav to success in business

### HEALTH IS A DUTY.

"A dying man can do nothing easy," were the last words of the immortal Franklin. A diseased man can do nothing well, are words of our own, quite as true.

If any thing should be well done, it should be the preparation which is needed to fit us for the exalted condition which has just been described, and to do it well, the highest health and the longest life should be sought by all. Such a preparation should be made under the most favorable of all possible conditions, and it is to no less an end, that this book has been conceived, to wit, to show the reader how health may be maintained, and how disease may be averted to the utmost limit of human life, that by the aid of health and length of days, the most perfect preparation possible may be made for the immortal existence beyond, and in this light, who shall deny that Health is a duty? Echo answers, 'Disease is a crime!'

"*Health and Disease.*"

